# The Emerging Roles of Autophagy-Related MicroRNAs in Cancer

**DOI:** 10.7150/ijbs.50773

**Published:** 2021-01-01

**Authors:** Chan Shan, Xinzhe Chen, Hongjing Cai, Xiaodan Hao, Jing Li, Yinfeng Zhang, Jinning Gao, Zhixia Zhou, Xinmin Li, Cuiyun Liu, Peifeng Li, Kun Wang

**Affiliations:** Institute of Translational Medicine, The Affiliated Hospital of Qingdao University, College of Medicine, Qingdao University, Qingdao 266021, China.

**Keywords:** Autophagy, MicroRNA, Post-translational regulation, Cancer

## Abstract

Autophagy is a conserved catabolic process involving the degradation and recycling of damaged biomacromolecules or organelles through lysosomal-dependent pathways and plays a crucial role in maintaining cell homeostasis. Consequently, abnormal autophagy is associated with multiple diseases, such as infectious diseases, neurodegenerative diseases and cancer. Currently, autophagy is considered to be a dual regulator in cancer, functioning as a suppressor in the early stage while supporting the growth and metastasis of cancer cells in the later stage and may also produce therapeutic resistance. MicroRNAs (miRNAs) are small, non-coding RNA molecules that regulate gene expression at the post-transcriptional level by silencing targeted mRNA. MiRNAs have great regulatory potential for several fundamental biological processes, including autophagy. In recent years, an increasing number of studies have linked miRNA dysfunction to the growth, metabolism, migration, metastasis, and responses of cancer cells to therapy. Therefore, the study of autophagy-related miRNAs in cancer will provide insights into cancer biology and lead to the development of novel anti-cancer strategies. In the present review, we summarise the current knowledge of miRNA dysregulation during autophagy in cancer, focusing on the relationship between autophagy and miRNAs, and discuss their involvement in cancer biology and cancer treatment.

## Introduction

Derived from Greek for 'self' (*auto*) and 'eating' (*phagy*), autophagy refers to the pathway by which cells transport cytoplasm to lysosomes for degradation 1. Currently, there are three known forms of autophagy, including chaperone-mediated autophagy, microautophagy and macroautophagy 2, 3, which differ in their physiological functions and delivery of substances to lysosomes 3. So-called 'autophagy' refers to macroautophagy, which is characterised by the formation of a double-layered envelope cellular structure under specific conditions that engulfs cytoplasm and organelles and transports them to lysosomes 4.

Autophagy is a conservative intracellular degradation mechanism that is maintained at low levels under normal cell conditions, while under stressful conditions, such as hunger, nutrient deficiencies, hypoxia, etc., autophagy can be rapidly upregulated 5, 6. Then, cellular components (such as long-lived proteins, protein aggregates, pathogens and damaged organelles) are wrapped and digested into small molecules for cell metabolism and recycling 5. However, the excessive activation of autophagy leads to cell death, which is defined as autophagic cell death and can be restrained by autophagy inhibitors 7. Abnormalities in autophagy can cause health problems, including inflammation, neurodegenerative diseases, cardiovascular diseases and cancer 1. Thus, identifying the molecular mechanisms of autophagic control is of crucial importance.

Non-coding RNAs, including long non-coding RNAs and circular RNAs, have been shown to participate in the regulation of autophagy 8, 9. Furthermore, the participation of microRNAs (miRNAs) in autophagy has also been investigated 10-13. MiRNAs are a class of non-protein-encoding RNA molecules that specifically bind to the 3'-untranslated region (3'-UTR) of target mRNAs, causing their degradation or protein translation inhibition to maintain optimal levels of the target protein 14. Functionally, miRNAs are involved in the regulation of a variety of cellular and molecular events, including cell proliferation, differentiation, metabolism and apoptosis 15. At present, approximately 2,000 miRNAs have been identified in humans, a number that continues to increase 16. The anomalous expression of miRNAs is associated with a variety of diseases, such as heart failure, muscular dystrophies, type 2 diabetes, Alzheimer's disease and cancer 17, with miRNAs contributing to the initiation and progression of several stages of cancer as well as resistance to anti-cancer therapy 18. Almost all malignant tumours exhibit differential expression of specific miRNAs between cancer and adjacent tissues 19, 20.

In the past few years, the results of a growing number of studies have emphasised the importance of miRNAs in autophagy regulation as well as the significance of autophagy in the biogenesis and function of miRNAs. In this review, we summarise recent advances in miRNA and autophagy and discuss the correlation between miRNA, autophagy and cancer.

## Molecular mechanisms of autophagy

**Figure 1:**
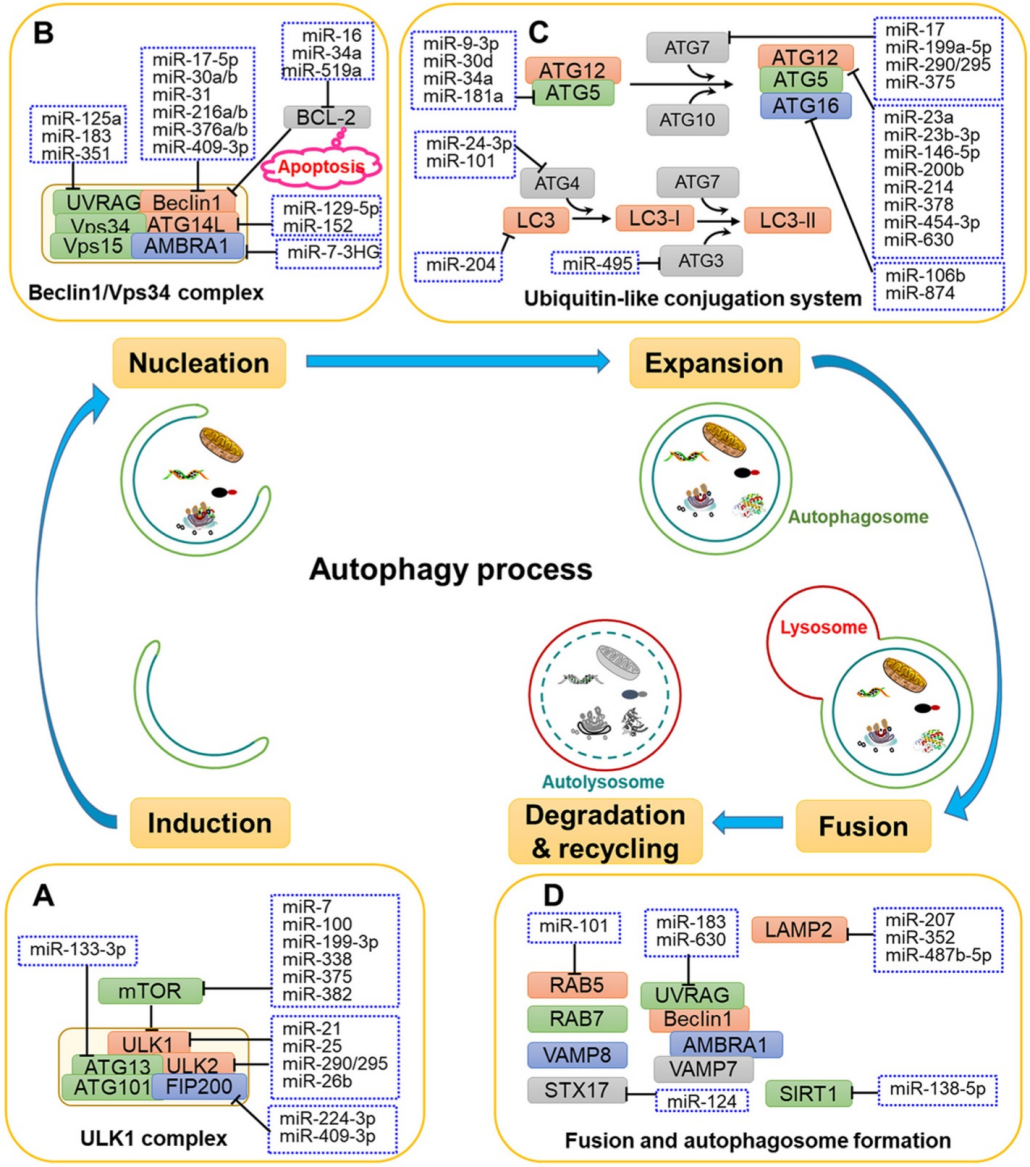
The roles of related miRNAs during the phases of autophagy. The core proteins regulated by miRNAs are shown in the schematic diagram. (A) Under some intracellular stress, such as hypoxia or starvation, autophagy is inducted by mTOR inactivation and activation of the downstream ULK1 complex. (B) Vesicle nucleation is driven by phosphorylation and activation of the Beclin 1/Vps34 complex. PI3P produced by the Beclin 1/Vps34 pathway recruits DFCP1 to provide a framework for membrane expansion. (C) During the membrane expansion step, two types of ubiquitin-like conjugation systems (the ATG5-ATG12 complex conjugated with ATG16L1, and LC3 conjugated with PE) are involved in the elongation and formation of autophagosomes. (D) The last crucial step is the fusion of autophagosomes with lysosomes to form autolysosomes for biomacromolecule degradation and recycling. GTPases, UVRAG, SNARE and other proteins are reported to be implicated in the fusion process.

The process of autophagy involves a series of stages and sequential membrane-remodelling events, including induction, vesicle nucleation, vesicle elongation, autolysosome maturation and lysosomal fusion, and degradation and recycling 21 (**Fig. 1**). The molecular mechanisms of autophagy are not fully understood, but it is thought that several autophagy-related genes (ATGs) and regulatory proteins are implicated in autophagy regulation.

### Induction

Target of rapamycin (TOR) kinase-containing protein complexes are the crucial autophagy regulators during the initiation stage. As shown in **Fig. 1A**, mammalian TOR (mTOR) is stimulated by intracellular stress and further activates the downstream serine/threonine Unc-51-like kinases 1 and 2 (ULK1 and ULK2). Subsequently, ULK1 and ULK2 form a ULK1 complex with ATG101, the family-interacting protein of 200 kDa (FIP200, the mammalian homologue of ATG17) and ATG13 22, thereby activating the initiation and nucleation process of autophagy.

### Vesicle nucleation

The second step of autophagy is vesicle nucleation. As shown in Fig. 1B, the ULK1 complex phosphorylates and activates the Beclin 1/Vps34 complex comprising Beclin 1, Vps34 (also called class III phosphatidylinositol 3-kinase, PIK3C3), ATG14L, Vps15 and the activating molecule in Beclin 1-regulated autophagy protein 1 (AMBRA1)^23^. The Beclin 1/Vps34 complex produces phosphatidylinositol 3-phosphatase (PI3P) to recruit the double-FYVE-containing protein 1 (DFCP1) that localises to the endoplasmic reticulum (ER) membrane, thereby providing a platform for expansion of the membrane and the formation of the omegasome. At present, the source of the membrane for autophagosomes has remained unclear, with potential sources including the ER membrane, the Golgi membrane, the plasma membrane and the mitochondria 24.

### Expansion

**Fig. 1C** shows the two types of ubiquitin-like conjugation systems that participate in the elongation of autophagosomes: the ATG5-ATG12 complex conjugated with ATG16L1 and microtubule-associated protein 1 light chain 3 (MAP1LC3, also called LC3) conjugated with lipid phosphatidylethanolamine (PE) 25. In the first system, the ubiquitin-like protein ATG12 covalently binds to ATG5 with the participation of the E1-like enzyme ATG7 and the E2-like enzyme ATG10. Next, the ATG5-ATG12 complex becomes localised to the autophagosome membrane to form a complex with ATG16, promoting the expansion of the autophagosome membrane 26. In the second system, the archetypal form of LC3 is cleaved by ATG4B to generate the cytosolic free LC3-I form 27. The E1-like enzyme ATG7, E2-like enzyme ATG3 and E3-like enzyme ATG12-ATG5-ATG16L1 are required for the linkage of LC3-I and PE to produce the LC3-PE complex, which is termed LC3-II 25. The high lipophilicity of PE promotes the attachment of LC3 to both faces of autophagosome membrane, where it plays a crucial role in membrane elongation and the selective degradation of cellular materials 28.

### Autolysosome maturation and lysosomal fusion

The last crucial step in the process of autophagy is the fusion of autophagosomes with lysosomes to form autolysosomes (**Fig. 1D**). Autophagosomes undergo several independent fusions to form amphisomes with late endosomes and subsequently forming autolysosomes with lysosomes 29. In the first step of autophagosome-lysosome fusion, the out autophagosomal membrane fuses with lysosomal membrane. The fusion process is finished by the degradation of the inner autophagosomal membrane and the exposure of autophagosome contents to the lumen of the lysosome 30. Several groups of proteins have been reported to be associated with the fusion process, including small GTPases, RAB proteins (RAB5 and RAB7), the UV radiation resistance-associated gene protein (UVRAG), and SNARE proteins (VAMP8 and STX17)^31^, ^32^. UVRAG is reported to play a role in the formation of membrane curvature, thus contributing to maturation of autolysosomes through its interaction with Beclin 1 33.

### Degradation and recycling

After fusion, superfluous or malfunctioning organelles and misfolded proteins harboured by autolysosomes are degraded by lysosomal acid hydrolases such as cathepsins (CTSs) into substrates for recycling and metabolism 34 (**Fig. 1D**). A variety of CTSs (such as CTSB, CTSC, CTSD, CTSL, and CTSS) have been implicated in biomacromolecule degradation 35.

## Interrelation between miRNAs and autophagy

Studies have demonstrated that miRNAs are crucial regulators in the autophagy process, participating in several steps of autophagy, including the upstream pathways that trigger autophagy, the subsequent developmental stages, and the later stage of degradation. Meanwhile, autophagy is also crucial in the generation and maintenance of miRNAs.

### Regulation of autophagy by miRNAs

MiRNAs have been shown to affect the expression of ATGs and related regulators to influence the phases of autophagy, including induction, vesicle nucleation, phagophore expansion, autolysosome maturation, lysosomal fusion and degradation (**Fig. 1 and Table 1**).

#### Regulation of autophagy induction by miRNAs

**Figure 2:**
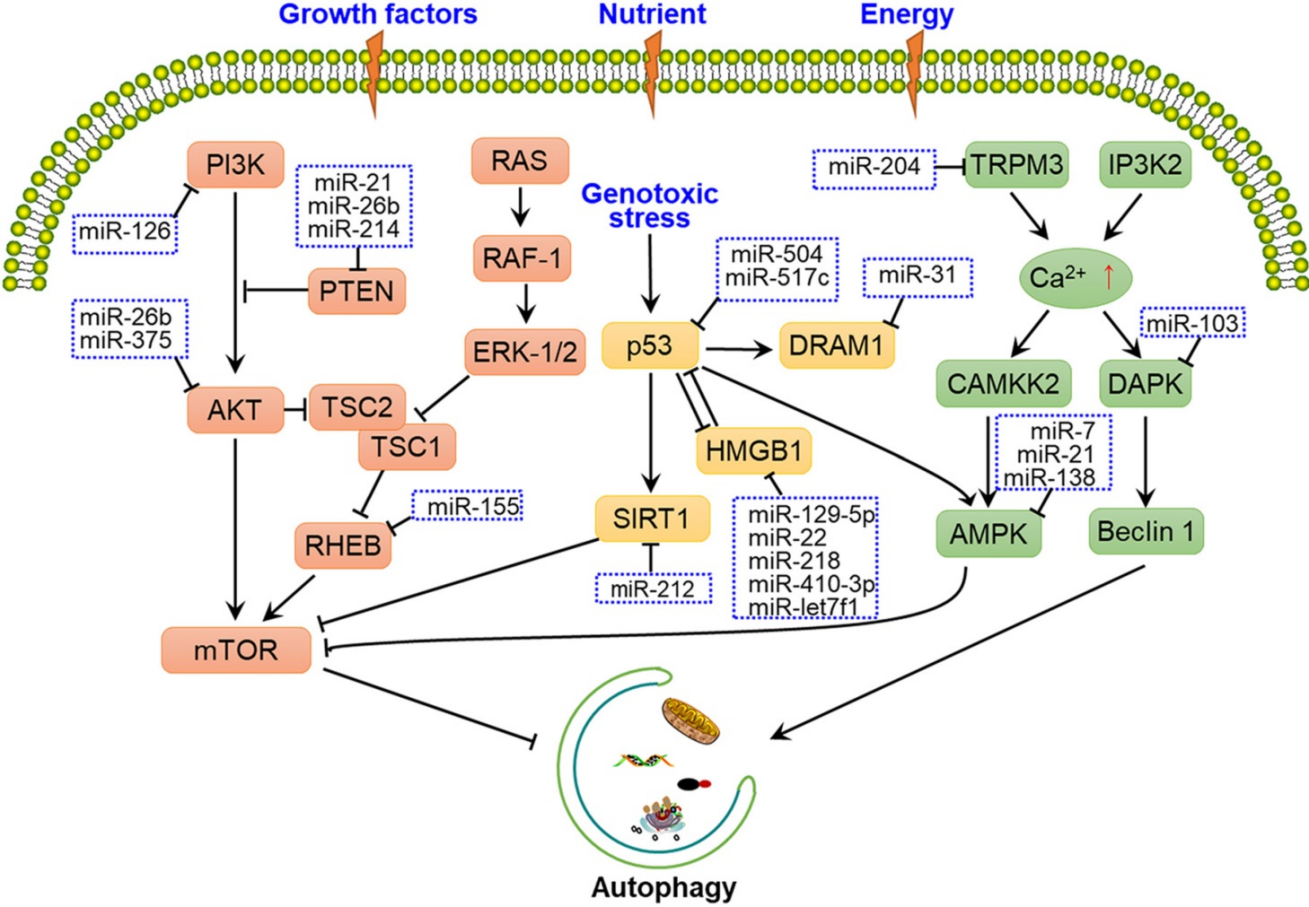
Overview of miRNAs involved in the regulation of autophagy-related signalling pathways. Several upstream nutrient and energy signals are associated with autophagy initiation, such as the PI3K-AKT-mTOR, TP53-mTOR, and Ca^2+^-AMPK-mTOR pathways. mTOR is the central protein in these signalling pathways, and miRNAs regulate autophagy-related signalling pathways by targeting crucial factors, such as PI3K, AKT, AMPK, and the ULK1 complex.

Several upstream nutrient and energy signals are involved in the regulation of autophagy induction, including the PI3K-AKT-mTOR, TP53-mTOR, and Ca^2+^-AMPK-mTOR pathways 36-38, with some miRNAs having been shown to alter these signals to affect phagophore induction (**Fig. 2**). MiR-21, miR-26b and miR-214 were shown to have a crucial role in autophagy by targeting PTEN through inhibition of the PI3K-AKT-mTOR pathway in cancer cells 39-41. While under genotoxic stress, TP53 and HMGB1 form complex to produce the reciprocal inhibitory effect, thereby regulate the downstream SIRT1-mTOR signalling. Some miRNAs, such as miR-517c, miR-129-5p, miR-218 and miR-212 have been shown to regulate autophagy through TP53-mTOR signalling 42-45. Some calcium-metabolizing enzymes, such as transient receptor potential melastatin 3 (TRPM3) and *Drosophila* inositol 1,4,5-trisphosphate kinase 2 (IP3K2) participate in the regulation of autophagy initiation through the Ca^2+^-AMPK-mTOR pathway. Hall et al. demonstrated that in clear cell renal cell carcinoma, miR-204 targets the TRPM3 channel, thereby promoting Ca^2+^ influx, the activation of CAMKK2, AMPK, and ULK1, and phagophore formation 36.

In addition to upstream signals, mTOR and ULK1 complex are directly regulated by miRNAs. For instance, miR-7, miR-99a, miR-338, miR-375 and miR-382 directly target mTOR to regulate autophagy, thereby disturbing the tumorigenic potential of cancer cells 46-50. Moreover, miR-21, miR-25, miR-290-295 cluster and miR-26b interact with the ULK1 complex and further disrupt autophagy initiation in a number of cancers 11, 51-54. FIP200 and ATG13 are also targeted by miRNAs, such as miR-133a-3p, miR-244-3p and miR-409-3p, thereby blocking FIP200- and ATG13-mediated autophagy 55-57 (**Fig. 1A and Fig. 2**).

#### Regulation of nucleation by miRNAs

Multiple miRNAs have been demonstrated to target the nucleation process to affect autophagy. As shown in **Fig. 1B**, miR-17-5p, miR-30a, miR-216, miR-376 and miR-409-3p bind to the 3'-UTR of Beclin 1 and inhibits its expression, causing the suppression of autophagy in different types of cancer cell 58, 59. The importance of Beclin 1 is also reflected in its involvement with two cellular events, autophagy and apoptosis, which is achieved by its association with BCL-2 60. Some miRNAs, such as miR-16, miR-519a and miR-34a, increase apoptosis and autophagy by interacting with BCL-2 61-63.

In addition, miRNAs targeting ATG14 and UVRAG also affect the autophagy process. For example, miR-129-5p and miR-152 adjust autophagy by targeting ATG14 and further affects the PI3K/AKT/mTOR signal pathway 64, 65. In addition, miR-183, miR-351 and miR-125a specifically target the 3'-UTR of UVRAG, which is involved in vesicle formation 66, 67.

#### Regulation of membrane expansion by miRNAs

Multiple miRNAs have been reported to participate in the ATG5-ATG12-ATG16L and LC3-PE conjugation systems to regulate the elongation of autophagosomes (**Fig. 1C**). For example, miR-9-3p, miR-30d, miR-34a and miR-181a are demonstrated to regulate autophagy in cancer cells through targeting ATG5 68-71. In addition, the E1-like enzyme ATG7 is targeted by miR-7, miR-199a-5p, miR-290-295 cluster and miR-375 in several kinds of cancer cells, respectively 49, 54, 72, 73. Moreover, miR-23a, miR-23b-3p, miR-146a-5p, miR-200b, miR-378 and miR-630 were reported to regulate autophagy by binding to ATG12 in different types of cancer cells 74-78. Moreover, miR-874 can decrease the expression of ATG16L, thereby inhibiting autophagy and sensitizing gastric cancer (GC) cells to chemotherapy 79.

MiR-204 has been shown to regulate LC3-II in IR-induced cardiomyocyte autophagy 80. In addition, ATG4 is reported to targeted by miR-24-3p and miR-101 in lung cancer and hepatocellular carcinoma cells, respectively 81, 82. Moreover, the E2-like enzyme ATG3 is targeted and regulated by miR-495 under starvation conditions 83. MiR-495 suppresses starvation-induced autophagy by inhibiting the conversion of LC3-I to LC3-II and reduces the number of autophagosomes.

#### Regulation of autolysosome maturation by miRNAs

The late maturation and fusion steps are regulated by multiple proteins, such as Rab5, SIRT1, UVRAG, STX17 and lysosomal associated membrane protein 2 (LAMP2) 84-86. As shown in **Fig. 1D**, miR-138-5p has been shown to target the 3'-UTR of sirtuin 1 (SIRT1), further regulating the SIRT1/FoxO1/Rab7 axis and inhibiting autophagy 87. Moreover, Huang and colleagues demonstrated that miR-124 downregulates STX17 expression by targeting the 3'-UTR to regulate retinoblastoma cell autophagy 88. Additionally, miR-207, miR-352 and miR-487b-5p directly target LAMP2 to affect the latter stage of lysosomal-autophagy flux in cortical neuronal cells 89, 90.

Thus, the study of miRNAs as regulators of autophagy-lysosomal-associated genes is important to elucidate the molecular mechanisms underlying the development of autophagy and provides insights for clinical applications.

### Regulation of miRNAs by autophagy

Autophagy is a process in which intracellular cytoplasmic substances are transported to lysosomes for degradation, and miRNAs are no exception. Considering that lysosomes contain multiple proteases and RNases, they may not only regulate RNA processes through the degradation of RNA-related proteins but could also affect RNA itself 91.

**Figure 3:**
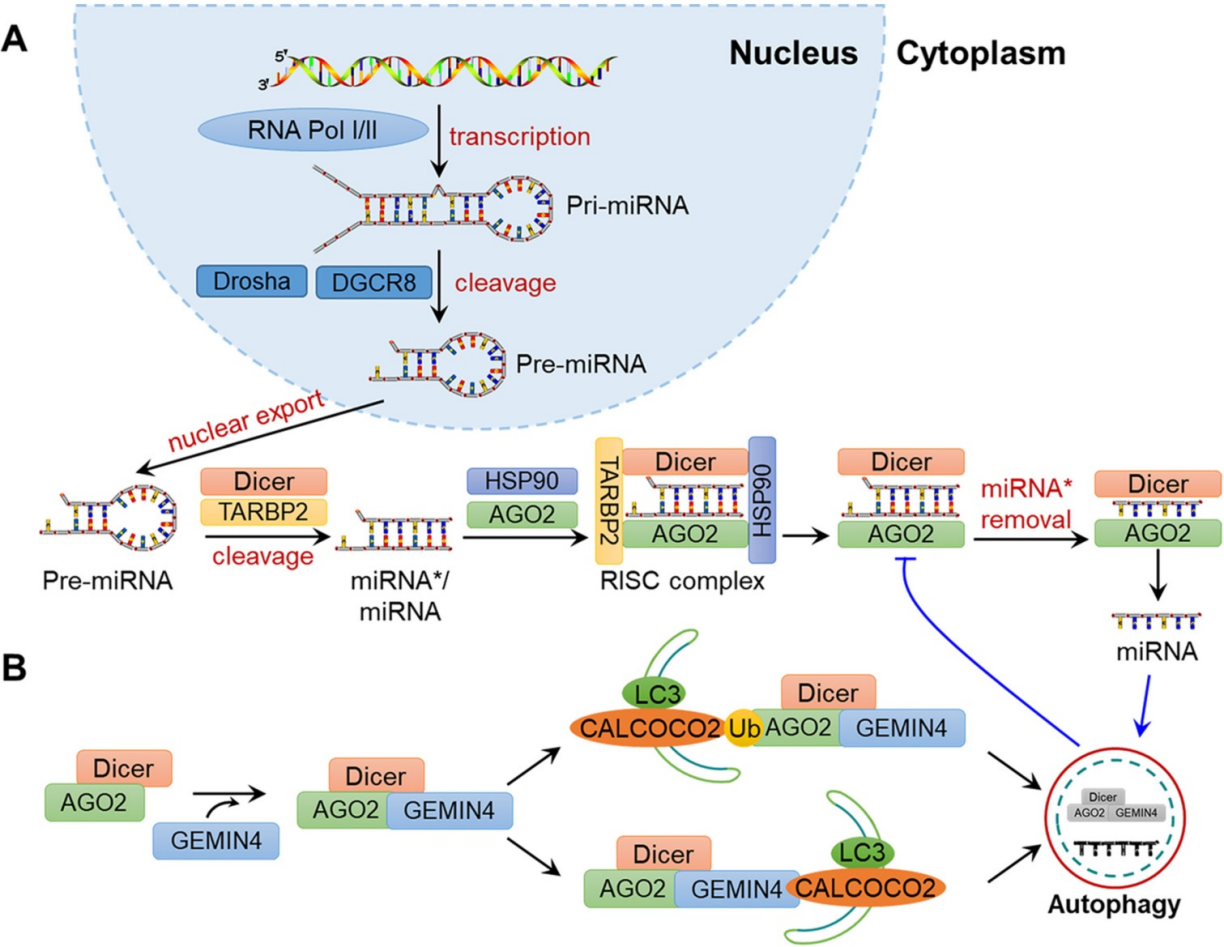
Schematic diagram of miRNA biosynthesis and regulation by autophagy. (A) Biosynthesis of miRNAs. In the nucleus, a pri-mRNA is transcribed under by RNA Pol Ⅰ/Ⅲ and further cleaved by Drosha and DGCR8 to generate pre-miRNA. After its nuclear export, Dicer cleaves the pre-miRNA into an miRNA*/miRNA duplex, which is then loaded to the groove of AGO2 to form the RISC complex. The miRNA then guides RISC to silence the target mRNA through mRNA cutting, transcriptional inhibition or deadenylation, while miRNA* is degraded. (B) Regulation of miRNA by autophagy. Dicer and AGO2 are associated with GEMIN4 to form the Dicer-AGO2-GEMIN4 complex. The Dicer-AGO2-GEMIN4 complex may interact with CALCOCO2 through either the ubiquitination of AGO2 or GEMIN4. Finally, Dicer and AGO2 are degraded by the autophagy-lysosome system, thereby inhibiting miRNA homeostasis. In addition, miRNA itself may be degraded by autophagy.

A variety of proteins are involved in the biosynthesis of miRNAs. As shown in **Fig. 3A**, pre-miRNAs exported from the nucleus are cleaved into double-stranded miRNA*/miRNA duplex by Dicer. Then, miRNA*/miRNA is transferred to the groove of RNA-induced silencing complex (RISC) component argonaute 2 (AGO2), and miRNA* becomes dissociated to generate mature miRNA. AGO2 can repress miRNA-targeted mRNAs through translational inhibition of those mRNAs and the promotion of mRNA decay. Gibbings et al. reported that AGO and Dicer can be degraded by autophagy 92. Specifically, the autophagy receptor CALCOCO2 is associated with AGO2 and Dicer in a GEMIN4-dependent manner, demonstrating the crucial effects of autophagy in miRNA biosynthesis and homeostasis (**Fig. 3B**).

In addition, miRNAs can also be directly degraded during autophagy (**Fig. 3B**). For example, Lan and colleagues demonstrated that miR-224 is selectively degraded by the autophagosome-lysosome system, and the downregulation of autophagy is negatively associated with the accumulation of miR-224 in hepatocellular carcinoma (HCC)^93^. Peng et al. suggested that CDKN1B promotes the degradation of miR-6981 through SQSTM1/p62-mediated autophagy, thereby resulting in a tumour-suppressive effect 94. On the whole, autophagy is altered under various types of cellular stress, infections and disease in mammals, which may influence the abundance, activity and function of miRNAs and provide new ideas for understanding the physiological and pathological processes in humans.

## Roles of autophagy-related miRNAs in cancer

Many miRNAs have been shown to participate in various stages of autophagy by regulating the expression of autophagy-related genes and proteins. These miRNAs are also involved in different stages of cancer development and progression, affecting cancer cell survival and proliferation, metabolism, oxidative stress, metastasis and deterioration, as well as resistance to radiation and chemotherapy. Indeed, many autophagy-related miRNAs have been recognised as diagnostic and prognostic biomarkers that may be useful as clinical therapeutic targets for cancer. In this section, we review the effects of miRNAs and autophagy and their interactions with respect to cancer biology as well as their associated responses to anti-cancer therapy.

### Cellular context-dependent functions of miRNAs in cancer

MiRNAs are a class of endogenous non-coding RNAs that are approximately 20-25 nucleotides in length and can recognise target mRNAs through complementary base pairing, directly silencing RISC to degrade target mRNA^95^. Recent research has revealed that miRNAs can serve as new targets for the treatment of diseases, especially in cancers.

A pioneering study by Calin and colleagues showed that miR-15 and miR-16 are deleted in chronic lymphocytic leukaemia, indicating that miRNAs may function as cancer suppressors 96. Subsequently, countless miRNAs have been demonstrated to participate in the pathogenesis of cancer. Currently, miRNAs are known to participate in various types of cancers, including lung cancer, breast cancer, melanoma, colorectal cancer (CRC), and leukaemia 97. The relationship between dysregulated miRNAs and tumorigenesis has been a hot topic in cancer biology investigations.

A single miRNA molecule can target tens to hundreds of different mRNAs, some of which may have opposite carcinogenic or tumour-suppressor functions. Thus, a specific miRNA may have the ability to be oncomiRNA as well as tumour-suppressive miRNA, depending on the cellular context 98. For example, miR-7 was reported to have hundreds of targets and have cellular context-dependent activities in cancer 99. On the one hand, miR-7 targets and downregulates tumorigenic factors in tumour-associated signalling pathways such as PA28γ, EGFR, PAK1, ACK1 and PIK3CD, demonstrating the crucial role of miR-7 in tumour suppression 100. On the other hand, elevated levels of miR-7 have been shown to be associated with tumour aggressiveness in cervical cancer (CC) and lung adenocarcinoma (LAC) 101, 102.

Regarding members of the miR-125 family, targets include pro-apoptosis genes (*P53INP1*,* TNFAIP3*,* Bmf*,* Puma*,* p53*, and *Bak1*), anti-apoptosis genes (*MUC1*,* Mcl-1*,* Bcl-w*, and *Bcl-2*), pro-proliferation genes (*c-Jun*,* ERBB2/3*,* VEGF-A*, and *E2F3*), metastasis-inhibiting genes (*STARD13*,* TP53INP1*, and *p53*), metastasis promoting genes (*MMP13*,* LIN28B*,* ARID3B*) and pro-differentiation genes (*IGF2*,* DICER1*,* LIN28A*,* CBFβ*, and *ARID3a*) 103. The balance of these oncogenes/tumour-suppressive genes in different cellular contexts leads to the oncogenic or tumour-suppressive effect of the miR-125 family in specific cancers. MiR-125b was reported to be upregulated in acute megakaryoblastic leukaemia and acute promyelocytic leukaemia, while in aggressive and indolent chronic lymphocytic leukaemia, miR-125b was observed to be downregulated 104-106.

Another example is miR-155, which in most instances is considered to function as an oncomiRNA, possessing oncogenic roles in cancers. MiR-155 has been to be overexpressed in several haematological and solid malignancies, such as Hodgkin's lymphoma, breast cancer, lung cancer, CC and thyroid carcinoma 98. Nevertheless, miR-155 was demonstrated to play a tumour-suppressive role in some tumours. For example, Qin et al. and Li et al. confirmed that miR-155 has a tumour-suppressive effect in ovarian cancer and GC 107, 108.

The controversial properties of miRNAs in different cancers suggest that the functions of miRNAs vary with respect to the pathogenesis and progression of cancer. Therefore, the underlying mechanisms that occur under different cellular contexts, requires further investigation.

### Dual roles of autophagy in cancer

Autophagy is an important biological phenomenon and is involved in regulating the balance between the synthesis, degradation and reuse of cellular material, thereby reducing the accumulation of intracellular waste and limiting abnormal mutations and genomic damage that may lead to cancer 109. In this respect, normal autophagy can be considered to be a suppressive mechanism in the early stage of cancer. Research has shown that deletions of the tumour-suppressor gene* Beclin 1* often occurs in breast, ovarian and prostate cancers, the loss of which causes a reduction of autophagy and the promotion of cellular proliferation 110, 111. Another example is Bif-1, a protein that interacts with Beclin 1 through UVRAG and positively regulates autophagy 33. Bif-1 knockout can lead to the inhibition of autophagy and enhance the development of cancer in mice. Taken together, these results establish the suppressive role of autophagy in cancer.

Conversely, in established tumours, autophagy can allow tumour cells to meet the high metabolic demands of continuous proliferation, thereby protecting tumour cells from metabolic stress-induced necrosis 112, which has been well illustrated for RAS-activating mutations. RAS activation leads to the upregulation of autophagy via the RAF/MEK/ERK pathway, which is associated with the regulation of mitochondrial metabolism 113. Indeed, researchers have demonstrated increased autophagy in a variety of RAS-activated cancer cells 114, 115. The inhibition of autophagy induces decreased glycolytic capacity, oxidative phosphorylation, and cell proliferation as well as increased apoptosis *in vitro* and *in vivo*.

Autophagy has important significance in the establishment and development of cancer. Thus, gaining a better understanding the cellular and functional correlation of autophagy in tumour microenvironment will help translate laboratory research into clinical applications.

### Involvement of miRNAs and autophagy in cancer biology

Many autophagy-related miRNAs participate in different biological events in cancer, including cancer proliferation, cellular metabolism, angiogenesis, cancer cell migration and metastasis, as well as responses to cancer therapy. More importantly, some autophagy-related miRNAs have been regarded as cancer biomarkers and anti-cancer targets. In this section, we summarise these autophagy-related miRNAs and their roles in cancer biology and anti-cancer applications (**Table 1**).

#### Cell survival and proliferation

Autophagy is crucial for the survival and growth of cancer cells, and miRNAs have been demonstrated to regulate autophagy and control cell growth and proliferation.

For instance, Guo and colleagues observed that miR-532-3p can bind to the 3'-UTR of RAB3IP, increasing the number of autophagosomes and LC3-II expression to inhibit cell viability and growth in GC 116. The overexpression of miR-375 has been shown inhibit cell proliferation and AKT/mTOR-dependent autophagy in GC both *in vitro* and *in vivo*, providing a potential therapeutic target for GC 49. Additionally, miR-423-3p overexpression was shown to activate autophagy and promote proliferation in GC cell lines and animal models 117. Thus, miR-423-3p may be a new biomarker and potential target for the treatment of GC. Moreover, Xu and colleagues reported that miR-1256 suppresses cell proliferation, thereby impairing autophagy in GC cells by targeting calcium-binding protein 39 (CAB39), an upstream regulator of the AMPK/mTOR pathway 118. In another study, miR-133b was observed to induce autophagy and inhibit GC cell proliferation by targeting polypyrimidine tract-binding protein 1 (PTBP1) 119.

Similarly, in NSCLC, Ye et al. demonstrated that miR-138 inhibits cell proliferation by suppressing the AMPK signalling pathway and increasing mTOR phosphorylation 120. Li et al. confirmed that miR-21 promotes the proliferation, migration and invasion of NSCLC and can induce autophagy by activating the AMPK/ULK1 pathway 11. Moreover, miR-18a-5p is regarded to function as a regulator of autophagy by targeting EGFR, thereby inducing proliferation inhibition and apoptosis activation in NSCLC cells 121.

Autophagy-related miRNAs have also been shown to adjust cellular proliferation in CC. MiR-519d-3p, which is downregulated in CC, can inhibit cell proliferation, cause cell cycle arrest and promote apoptosis and autophagy by targeting hypoxia-inducible factor-2α (HIF-2α) 122. In addition, Wan et al. demonstrated that miR-155 is an autophagy inducer that downregulates mTOR signalling, thereby decreasing cellular proliferation and causing cell cycle arrest in CC cells 123. Moreover, Capizzi et al. showed that miR-7-3HG is an autophagy inhibitor that targets the 3'-UTR of AMBRA1 in CC, where inhibiting this miRNA reduced the proliferation and increased apoptosis of cancer cells 124.

Numerous studies have investigated the effects of autophagy-related miRNAs in regulating the survival and proliferation of cancer cells. For example, miR-145-3p overexpression was shown to inhibit the proliferation of osteosarcoma cells while activating apoptosis and autophagy by downregulating HDAC4 125. In addition, the downregulated miRNA, miR-30d, was demonstrated to bind the 3'-UTRs of ATG5, PI3K and Beclin 1, thereby negatively regulating autophagy, inhibiting the proliferation and viability of colon cancer cells 69. MiR-26b was observed to be upregulated in laryngeal carcinoma patients 52. Interestingly, miR-26b downregulation was shown to activate autophagy in a ULK2- and PTEN/AKT-dependent manner and could further inhibit the proliferation and induce the apoptosis of laryngeal carcinoma cells. In urothelial carcinoma, miR-331-3p was demonstrated to reduce cell proliferation by targeting nucleus accumbens-associated protein 1 (NACC1) 126.

#### Cell metabolism

Cancer cells undergo major changes in glucose, amino acid and fatty acid metabolism to maintain survival in a stressful microenvironment. As autophagy is an intracellular recycling process that maintains the levels of metabolites and biosynthetic intermediates under starvation or other stress conditions, it is an important mechanism for cancer cell metabolic adaptation 127. Importantly, multiple autophagy-related miRNAs are involved in the metabolic regulation and stress responses of cancer cells. For example, Gu and colleagues reported that pancreatic cancer cells can use autophagy to meet the requirement of glycolysis 47. Specifically, miR-7 inhibits autophagy by upregulating the LKB1-AMPK-mTOR pathway, reducing the intracellular glucose supply for glycolysis. MiR-126 was demonstrated to induce autophagy in malignant mesothelioma cells by downregulating the insulin receptor substrate-1 (IRS1) signalling pathway, further suppressing glucose uptake and leading to energy exhaustion and the AMPK-dependent phosphorylation of ULK1 128. Additionally, Chen et al. showed that the miR-290-295 cluster can enhance the resistance of melanoma cells to glucose starvation in an ATG7/ULK1-dependent manner, inhibiting autophagic cell death caused by glucose starvation 54.

Regarding the regulation of lipid metabolism, miR-494 can increase multilamellar bodies and lipid droplets formation that is accompanied by autophagy activation in renal cancer cells 129. Additionally, miR-126 was demonstrated to induce autophagy by stimulating lipid droplet accumulation in an HIF-1α-dependent manner in malignant mesothelioma cells 128. Autophagy-related miRNAs have also been shown to be involved in glutamine metabolism in cancer. Zhang and colleagues demonstrated that GC cells could supplement glutaminolysis through autophagy, whereas miR-133-3p can block this process by targeting GABA type A receptor-associated protein like 1 (GABARAPL1) and ATG13 55.

Although these basic research studies have demonstrated that autophagy-related miRNAs are involved in the regulation of cancer cell metabolism, additional work is required to comprehensively elucidate the mechanisms underlying this metabolic regulatory network.

#### Angiogenesis

Autophagy is essential for endothelial cell function and angiogenesis 130. Recently, some autophagy-related miRNAs have been shown to affect the survival, growth and spread of endothelial cells, thereby directly affecting cancer angiogenesis. For example, the inhibition of miR-195, which targets GABARAPL1, was shown to promote the proliferation and migration of endothelial progenitor cells (EPCs) as well as cancer angiogenesis under hypoxia 131. Furthermore, miR-212 was demonstrated to negatively regulate autophagy by targeting SIRT1, thereby leading to the inhibition of angiogenesis and cell senescence in prostate cancer cells 44. Wang and colleagues showed that miR-210 expression is associated with vascular endothelial growth factor (VEGF) levels in schwannoma cells 132. Importantly, hypoxia was shown to induce the demethylation of miR-210 and affect HIF-1α/VEGF-mediated angiogenesis. This result led to a model in which hypoxia enhances miR-210 expression and promotes autophagy, thereby increasing angiogenesis in schwannoma. The results of these studies indicate the role of miRNAs and autophagy in regulating endothelial cell homeostasis and tumour angiogenesis *in vitro*, although additional research is needed to establish their relevance *in vivo*.

#### Cancer cell migration and metastasis

The link between cell autophagy and metastasis affects cancer cell mobility, invasion and metastasis, with some studies having suggested that autophagy-related miRNAs are associated with cancer migration and metastasis 133.

A number of studies have shown that miRNAs regulate migration and metastasis by inhibiting autophagy. For instance, miR-338-5p is positively associated with the stage, metastasis and poor prognosis in primary CRC by directly targeting PIK3C3 pathway, resulting in both migration and invasion *in vivo* 134. In cutaneous melanoma, the downregulated miRNA, miR-23a, suppressed the invasion and migration of cells by inhibiting ATG12-mediated autophagy 74. Moreover, miR-378 overexpression directly reduces ATG12 levels, inhibiting autophagy and resulting in enhanced migration and invasion in CC 77. Yuan et al. observed that miR-375 inhibits autophagy in GC by targeting AKT/mTOR and is negatively associated with cellular migration capacity 49. Similarly, miR-517c was demonstrated to inhibit autophagy and reduce glioblastoma cell migration depending on TP53 expression 42. Additionally, miR-454-3p was shown to inhibit the migration and invasion of glioma cancer cells by targeting ATG12 135.

In contrast, in some cases, miRNAs regulate cancer migration and metastasis by activating autophagy. For example, miR-382, promotes apoptosis and autophagy in esophageal squamous cell carcinoma (ESCC) by inhibiting mTOR and 4E-BP1 50, and the overexpression of miR-382 was shown to suppress the migration, invasion and epithelial-mesenchymal transition (EMT) of ESCC cells. In NSCLC, miR-18a-5p was reported to promote autophagy by targeting interferon regulatory factor 2 (IRF2) to increase apoptosis while inhibiting cellular migration 121. MiR-524-5p, a downregulated miRNA in papillary thyroid carcinoma, was shown to promote autophagy and inhibit cell viability, invasion, migration and apoptosis by targeting ITGA3 and FOXE1 in papillary thyroid cancer 136.

### Autophagy-related miRNAs and their response to cancer therapy

Radiotherapy and chemotherapy are currently the most important anti-cancer strategies. However, the therapeutic resistance of cancer cells is a major challenge in cancer treatment. Autophagy induced by therapeutants has been demonstrated to be related to the response, resistance or death of cancer cells, and increasing evidence suggests that many miRNAs are involved in the regulation of autophagy processes caused by anti-cancer therapies 23.

#### Response to radiotherapy

Radiotherapy is a standard treatment for various cancers and involves damaging cancer cells with ionising radiation. The mechanisms of radiation therapy include the production of oxygen radicals, which damage important organelles like mitochondria, and damage to biological macromolecules like DNA 137. Indeed, autophagy is an important factor that influences the effects of radiotherapy and cellular responses 138. Consequently, autophagy-related miRNAs are capable of regulating the response of cancer cells to radiotherapy. In this section, we summarise the effects of autophagy-related miRNAs on cancer radiotherapy (**Table 2**).

For example, miR-17-5p was demonstrated to target Beclin 1 and inhibit autophagy in glioma cells 59. In a mouse model, miRNA-17-5p overexpression sensitised the response of tumour tissues to irradiation, while miR-23b was shown to suppress irradiation-induced autophagy by targeting ATG12 in pancreatic cancer cells. Interestingly, miR-23b overexpression was observed to increase the sensitivity of pancreatic cancer cells to irradiation 139.

In contrast, in some cellular contexts, autophagy tends to reduce the sensitivity of cancer cells to radiation and can lead to resistance to radiation-induced cell death. For example, in prostate cancer cells, miR-32 was demonstrated to activate autophagy by targeting the tumour-suppressor gene DAB2 interacting protein (DAB2IP), thereby enhancing cell survival and decreasing sensitivity during radiation treatment 140. Similarly, miR-138-5p was observed to enhance radiation-induced autophagy by targeting EIF4EBP1 in nasopharyngeal carcinoma 141. Moreover, miR-301a/b was reported to induce autophagy and radioresistance by decreasing NARG2 expression in prostate cancer cells 142.

At present, the relationships and boundaries between protective and pro-survival autophagy, cellular apoptosis and necrosis remain unclear and requires a better understanding of the molecular mechanisms. Nonetheless, the regulation of autophagy in a cellular context-dependent manner may alter the response of cancer cells to radiotherapy.

#### Response to chemotherapy

A variety of chemotherapy drugs have been shown to induce the autophagy of cancer cells, and autophagy-related miRNAs have been reported to affect cancer cell susceptibility to anti-cancer drugs. **Table 3** summarises the effects of autophagy-related miRNAs on cancer chemotherapy.

For instance, miR-101 and miR-199a-5p were demonstrated to intensify cisplatin-induced cell death through inhibition of autophagy in HCC cells 73, 82. Similarly, cytoprotective autophagy was suppressed while the toxic effects of cisplatin were increased in GC cells when miR-148a-3p and miR-181a were overexpressed 71, 143. Moreover, in lung cancer cells, miR-146a-5p, miR-17-5p, miR-200b and miR-487b-5p have been shown to inhibit autophagy and enhance cellular chemosensitivity to cisplatin, paclitaxel, docetaxel and to temozolomide, respectively 76, 90, 144, 145.

On the other hand, miRNAs confer chemoresistance to anti-cancer agents by activating autophagy. For example, cisplatin-treated NSCLC cells were shown to become chemoresistant via miR-425-3p-facilitated autophagy activation in an AKT1-dependent manner 146. Similarly, in colon cancer cells, miR-338-3p was shown to promote 5-Fu resistance by inhibiting mTOR and activating autophagy 147. Moreover, miR-15a/16 was demonstrated to attenuate the phosphorylation of mTORC1 and p70S6K and to enhance camptothecin-induced autophagy in CC HeLa cells, thereby contributing to the efficacy of chemotherapy 148.

Indeed, autophagy-related miRNAs are involved in the response to chemotherapy in a variety of cancers. With the in-depth elucidation of mechanisms associated with autophagy-related miRNAs and cancer chemotherapy, additional autophagy-related miRNAs may become anti-cancer targets and prognostic biomarkers.

## Conclusion and prospection

Autophagy is an evolutionary mechanism that involves the recycling of biological macromolecules and organelles 5. MiRNAs participate in many biological processes and play a crucial role in the regulation of autophagy in cancer. The impact and number of studies on miRNAs and autophagy in cancer have continued to increase. However, autophagy and miRNA research is in its infancy, and requires further investigation, including with respect to the paradoxical effects of autophagy and miRNAs on cancer and the development of additional research methods and applications.

The role of miRNA-mediated autophagy in cancer remains controversial, and whether miRNA-regulated autophagy is a survival or death mechanism for cancer cells remains unknown 112. In addition, miRNAs themselves appear to exert bilateral regulation in cancer 98. Thus, the unclear role of autophagy and the dual roles of miRNAs in cancer complicate the associated regulatory mechanisms.

MiRNAs regulate gene expression before observable changes in protein levels, making autophagy-related miRNAs potential early autophagy markers that are superior to LC3 and SQSTM1 for monitoring autophagy 149. In recent years, a variety of miRNA detection methods with high resolution have been developed. For example, Guk et al. proposed a novel strategy involving the self-circulation of molecular beacon circuits and miRNAs hybridization for miRNA detection 150. In addition, Li and colleagues developed a novel and sensitive fluorescence polarization miRNA detection method with a detection limit of 0.001 nM 151. Moreover, Jin et al. proposed a sensitive miRNA detection method using Chlorella virus DNA ligase that is at least 40-fold more sensitive than conventional methods and could detect individual miRNAs that differ by only one nucleotide 152. Additionally, because studies on the effects of a specific miRNA on a single autophagy gene is often one-sided, multidisciplinary and comprehensive public databases should be used to assess the regulation of multiple genes and steps in the complex autophagy network by miRNAs.

The regulation of autophagy by miRNAs can affect the sensitivity of cancer to radiotherapy and chemotherapy. However, considering the development of cell resistance caused by autophagy during treatment, it is not appropriate to simply abandon cell survival in favour of cell death. Indeed, autophagy may be the link between cell survival and death, and these dysregulated autophagy-related miRNAs could serve as potential anti-cancer targets. Therefore, the biosafety and reliability of miRNA-based therapeutic strategies should receive widespread attention, including with respect to the exact role of miRNAs in specific autophagy steps, techniques for effectively delivering miRNA mimics *in vivo*, and therapeutic strategies based on miRNA off-target effects.

Autophagy-related miRNAs have great potential for use in the diagnosis, treatment, and prognosis evaluation of cancer. Thus, an in-depth exploration of autophagy and miRNA as well as related applications should be a major objective for scientists.

## Figures and Tables

**Table 1:** Autophagy-associated miRNAs in cancer

Effect on autophagy	Name	Dysregulation	Autophagy-related target	Types of cancer cell line (cancer tissue)	Ref.
Inhibition	miR-7	N.D.	LKB1-AMPK-mTOR	AsPC-1, BxPC-3 and SW1990 (pancreatic cancer)	47
	miR-7-3HG	N.D.	AMBRA1	Hela (cervical cancer)	124
	miR-9-3p	N.D.	ATG5	TT and MZ-CRC-1 (medullary thyroid carcinoma)	68
	miR-10b	up-regulated	Bim/TFAP2C	U251, LN-308, and U373 (glioblastoma)	153
	miR-15a/16	downregulated	BCL-2	A549 (lung cancer)	148
	miR-17	up-regulated	ATG7	T98G and U373-MG (glioblastoma)	72
	miR-17-5p	downregulated	Beclin 1	A549 (lung cancer)	144
	miR-23a	downregulated	ATG12	WM35, WM793, 451LU, A2058 and A375 (melanoma)	74
	miR-26b	up-regulated	ULK2/AKT/PTEN	Hep-2 (laryngeal carcinoma)	52
	miR-29b	downregulated	PSME4	AMCL1, AMCL2 (myeloma)	154
	miR-30a	downregulated	Beclin 1	MG-63 (osteosarcoma)	155
	miR-30d	downregulated	ATG5	HCT15, HCT116, HT-29, DLD-1 and SW480 (colon cancer)	69
	miR-34a	downregulated	ATG5	SH-SY5Y and SK-N-SH (neuroblastoma)	70
	miR-101	downregulated	EZH2	HepG2 (liver cancer)	156
	miR-106b	N.D.	ATG16L1	HCT116 (colorectal cancer)	157
	miR-124/144	downregulated	PIM1	DU145 and PC3 (prostate cancer)	158
	miR-133-3p	downregulated	GABARAPL1/ATG13	BGC-823, SGC-7901, MGC-803, MKN-45, HGC-27 and AGS (gastric cancer)	55
	miR-138	downregulated	AMPK-mTOR	A549 and Calu-3 (lung cancer)	120
	miR-140-5p	downregulated	SMAD2	HCT116, RKO, and SW480 (colorectal cancer)	159
	miR-152	downregulated	ATG14	A2780/CP70, SKOV3/DDP (ovarian cancer)	65
	miR-183	up-regulated	UVRAG	HCT116 and HT29 (colorectal cancer)	160
	miR-195	up-regulated	GABARAPL1	Endothelial progenitor	131
	miR-204	N.D.	LC3	786-O, A498, and Caki-1 (kidney cancer)	161
	miR-212	downregulated	SIRT1	LNCaP (prostate cancer)	44
	miR-224-3p	up-regulated	FIP200	HeLa, SiHa, C33A (cervical cancer)	56
	miR-290-295 cluster	up-regulated	ATG7/ULK1	B16F1 (melanoma)	54
	miR-338	downregulated	mTOR	Siha, HeLa, C33 A and Me180 (cervical cancer)	48
	miR-338-5p	up-regulated	PIK3C3	SW480 and HCT116 (colorectal cancer)	134
	miR-340	downregulated	ROCK1	U373, U87 (glioblastoma)	162
	miR-375	downregulated	AKT/mTOR	MKN-45 and GT3TKB (gastric cancer)	49
	miR-378	up-regulated	ATG12	HeLa and C-33A (cervical cancer)	77
	miR-454-3p	downregulated	ATG12	U251, U87 and LN229 (glioma cancer)	135
	miR-502	downregulated	RAB1B	HCT116 (colorectal cancer)	163
	miR-517c	N.D.	TP53	U87 and U251 (glioblastoma)	42
	miR-519a	downregulated	BCL-1	BEAS-2B (lung cancer)	62
	miR-532-3p	up-regulated	RAB3IP	AGS, MKN45, BGC823, SGC7901, MGC803, and MKN28 (gastric cancer)	116
	miR-630	N.D.	ATG12/UVRAG	JHU-029 (squamous cell carcinoma)	78
	miR-638	up-regulated	TP53INP2	SK-Mel-28 and SK-Mel-147 (melanoma)	164
	miR-874	downregulated	ATG16L1	SGC7901, BGC823 SGC7901, BGC823 and AGS (gastric cancer)	79
	miR-1256	downregulated	CAB39	MKN45, MGC803, AGS, HGC27, BGC823 and SGC7901(gastric cancer)	118
Activation	miR-18a-5p	up-regulated	EGFR	A549, H23, H1299 and H1650 (lung cancer)	121
	miR-20a	up-regulated	THSB2	SiHa and HeLa (cervical cancer)	165
	miR-21	up-regulated	AMPK/ULK1	A549 (lung cancer)	11
	miR-99a	downregulated	mTOR	MCF‐7 and MDA‐MB‐231 (breast cancer)	46
	miR-100	downregulated	IGFR1/mTOR	HepG2, Huh7 (liver cancer)	166
	miR-126	downregulated	IRS1	H28 (sarcomatoid malignant mesothelioma)	128
	miR-133b	downregulated	PTBP1	MKN-1, MKN-45 and KATO-III (gastric cancer)	119
	miR-145-3p	downregulated	HDAC4	U2OS and MG-63 (osteosarcoma)	125
	miR-155	up-regulated	RHEB/RICTOR/RPS6KB2	CNE (nasopharyngeal cancer) and HeLa (cervical cancer)	123
	miR-155-3p	N.D.	CREBRF	U251 and T98G (glioblastoma)	167
	miR-210	up-regulated	VEGF	RT4-D6P2T (schwannoma)	132
	miR-382	downregulated	mTOR	Eca109 and Het-1A (esophageal squamous cell carcinoma)	50
	miR-423-3p	up-regulated	Bim	BGC823, MGC803, SGC7901, and MKN45 (gastric cancer)	117
	mR-494	N.D.	LC3	769-P (renal cancer)	129
	miR-519-3p	downregulated	N.D.	HeLa (cervical cancer)	122
	miR-524-5p	downregulated	ITGA3	TPC-1, K1, and NPA papillary (thyroid carcinoma)	136

**Table 2:** Effects of autophagy-related miRNAs on cancer radiotherapy

Effect on autophagy	Name	Dysregulated	Autophagy-related target	Effect on radiotherapy	Tested cell line (tissue origin)	Ref.
Inhibition	miR-17-5p	up-regulated	Beclin 1	radiosensitivity	U87 (glioblastoma)	59
	miR-21	up-regulated	PTEN	radiosensitivity	Hela, Siha(cervical cancer)	168
	miR-23b	downregulated	ATG12	radiosensitivity	BxPC3, PANC-1 (pancreatic cancer)	139
	miR-31	downregulated	Beclin 1, ATG, DRAM	radiosensitivity	Primary cultured cells (colorectal cancer)	169
	miR-93	up-regulated	Beclin 1, ATG5, ATG4B, SQSTM1	radiosensitivity	U87 (glioblastoma)	170
	miR-101	downregulated	STMN1	radioresistance	CNE-2, 5-8F (nasopharyngeal carcinoma)	171
	miR-129-5p	downregulated	HMGB1	radiosensitivity	MCF-7, MDA-MB-231 , BT549, BT474 (breast cancer)	43
	miR-183-5p	up-regulated	ATG5	radiosensitivity	Caco-2 (colon cancer)	172
	miR-200c	downregulated	UBQLN1	radiosensitivity	MDA-MB-231, BT549 (breast cacer)	173
	miR-214	downregulated	ATG12	radiosensitivity	SW480, HCT116 (colorectal cancer)	174
	miR-216b	up-regulated	Beclin 1	radioresistance	PANC-1 (pancreatic cancer)	175
	miR-450a-5p	downregulated	DUSP10	radioresistance	ECA (esophageal cancer)	176
Activation	miR-138-5p	downregulated	EIF4EBP1	radiosensitivity	HONE1, HK1 (nasopharyngeal carcinoma)	141
	miR-301a/b	up-regulated	NDRG2	radioresistance	LNCaP (prostate cancer)	142
	miR-1246	up-regulated	mTOR	radioresistance	A549, PC9 (lung cancer)	177
	miR-4673	up-regulated	CDK-18	radioresistance	SKBR3 (breast cancer)	178

**Table 3:** Effects of autophagy-related miRNAs on cancer chemotherapy

Effect on autophagy	MiRNAs	Dysregulation	Autophagy-related target	Effect on chemotherapy	Agent	Tested cell line (tissue origin)	Ref.
Inhibition	miR-let7f1	N.D.	HMGB1	chemosensitivity	Cisplatin	D425, UW228 (medulloblastoma)	179
	miR-30a	downregulated	Beclin 1	chemoresistance		HeLa, MCF-7, HepG2, HepS	180
	miR-101	N.D.	STMN1, RAB5A, ATG4D, mTOR	chemosensitivity		HepG2 (liver cancer)	82
	miR-146a-5p	up-regulated	ATG12	chemoresistance		A549 (lung cancer)	76
	miR-148a-3p	downregulated	AKAP1	chemoresistance		BGC823 (gastric cancer)	143
	miR-152	downregulated	ATG14	chemoresistance		A2780/CP70 (ovarian cancer)	65
	miR-181a	N.D.	ATG5	chemosensitivity		SGC7901 (gastric cancer)	71
	miR-199a-5p	downregulated	ATG7	chemoresistance		Huh7, HepG2 (hepatocellular carcinoma)	73
	miR-205	N.D.	RAB27A LAMP3	chemoresistance		DU145, PC-3 (prostate cancer)	181
	miR-409-3p	downregulated	FIP200	chemosensitivity		OV-1063 (ovarian cancer)	182
	miR-416a	up-regulated	CHOP	chemoresistance		A549, H446(lung cancer)	183
	miR-17-5p	downregulated	Beclin 1	chemosensitivity	Paclitaxel	A549-T24 (lung cancer)	144
	miR-216b	downregulated	Beclin 1	chemosensitivity		A549, Calu-3 (lung cancer)	184
	miR-218	downregulated	HMGB1	chemoresistance		RL95-2 (endometrial carcinoma)	45
	miR-143	downregulated	ATG2B	chemosensitivity	Doxorubicin	SAOS, U2OS (osteosarcoma)	185
	miR-223	downregulated	FOXO3a	chemoresistance		HepG2, Huh7, SNU387, SNU449 (hepatocellular carcinoma)	186
	miR-34a	downregulated	Smad4	chemoresistance	Oxaliplatin	HT29 (colorectal cancer)	187
	miR-409-3p	downregulated	Beclin 1	chemosensitivity		Lovo Oxa R(colon cancer)	182
	miR-17	up-regulated	ATG7	chemosensitivity	Temozolomide	U373 (glioma)	72
	miR-21	up-regulated	PTEN	chemosensitivity	Sorafenib	Huh7, HepG2 (liver cancer)	39
	miR-22	N.D.	BTG1	chemosensitivity	5-Fu	SW620 (colorectal cancer)	188
	miR-25	N.D.	ULK1	chemosensitivity	Isoliquiritigenin	MCF-7 (breast cancer)	53
	miR-30b	N.D.	Beclin 1	chemosensitivity	Imatinib	K562 (CML)	189
	miR-101	N.D.	STMN1, RAB5A, ATG4D	chemosensitivity	Etoposide	MCF-7 (breast cancer)	190
	miR-143	downregulated	GABARAPL1	chemosensitivity	Qercetin	AGS, MKN28 (gastric cancer)	191
	miR-200b	N.D.	ATG12	chemosensitivity	Docetaxel	SPC-A1, H1299 (lung cancer)	145
	miR-375	N.D.	ATG7	chemoresistance	Fulvestrant	MCF-7 (breast cancer)	192
	miR-410-3p	downregulated	HMGB1	chemoresistance	Gemcitabine	MiaPaCa2, PANC-1 (pancreatic cancer)	193
	miR-487b-5p	up-regulated	LAMP2	chemoresistance	Temozolomide	A549, H1299 (lung cancer)	90
Activation	miR-125b	downregulated	Foxp3	chemosensitivity	Cisplatin	WRO, FRO(thyroid cancer)	194
	miR-425-3p	up-regulated	AKT1	chemoresistance		A549 (lung cancer)	146
	miR-193b	up-regulated	STMN1	chemosensitivity	5-Fu	KYSE450 (oesophageal cancer)	195
	miR-338-3p	up-regulated	mTOR	chemoresistance		HCT116, HT29 (colon cancer)	147
	miR-15a/16	N.D.	Rictor	chemosensitivity	Camptothecin	Hela (cervical carcinoma)	148
	miR-16	downregulated	Bcl-2	chemoresistance	Paclitaxel	A549-T24 (lung cancer)	63
	miR-30a	downregulated	Beclin 1	chemosensitivity	Sorafenib	786-0, A489 (renal carcinoma)	196

## Abbreviations

3'-UTR: 3'-untranslated region
AGO: argonaute
AMBRA1: the activating molecule in Beclin 1-regulated autophagy protein 1
ATG: autophagy-related gene
CAB39: calcium-binding protein 39
CC: cervical cancer
CRC: colorectal cancer
CTS: cathepsin
DAB2IP: the tumour-suppressor gene DAB2 interacting protein
DFCP1: the double-FYVE-containing protein 1
EMT: epithelial-mesenchymal transition
EPC: endothelial progenitor cell
ER: endoplasmic reticulum
ESCC: esophageal squamous cell carcinoma
FIP200: the family-interacting protein of 200 kDa
GABARAPL1: GABA type A receptor-associated protein like 1
GC: gastric cancer
HCC: hepatocellular carcinoma
HIF-2α: hypoxia-inducible factor-2α
IRF2: interferon regulatory factor 2
IRS1: insulin receptor substrate-1
LAC: lung adenocarcinoma
LAMP2: lysosomal associated membrane protein 2
MAP1LC3: microtubule-associated protein 1 light chain 3
MiRNA: microRNA
mTOR: mammalian TOR
NACC1: nucleus accumbens-associated protein 1
NSCLC: non-small cell lung cancer
PE: phosphatidylethanolamine
PI(3)KC3: the class III phosphatidylinositol 3-kinase
PI3P: phosphatidylinositol 3-phosphatase
PTBP1: polypyrimidine tract-binding protein 1
PTEN: gene of phosphate and tension homology deleted on chromsome ten
RISC: the RNA-induced silencing complex
SIRT1: sirtuin 1
STX17: Syntaxin 17
THBS2: thrombospondin 2
TOR: target of rapamycin
ULK1: Unc-51-like kinases 1
ULK2: Unc-51-like kinases 2
UVRAG: the UV radiation resistance-associated gene protein
VAMP3: vesicle-associated membrane protein 3
VEGF: vascular endothelial growth factor
